# Factors Influencing Nonunion and Fracture Following Biological Intercalary Reconstruction for Lower‐Extremity Bone Tumors: A Systematic Review and Pooled Analysis

**DOI:** 10.1111/os.13546

**Published:** 2022-10-20

**Authors:** Siyi Huang, Hongfei Li, Zhili Xing, Tao Ji, Wei Guo

**Affiliations:** ^1^ Musculoskeletal Tumor Center Peking University People's Hospital Beijing China; ^2^ Key Laboratory for Musculoskeletal Tumor of Beijing Beijing China; ^3^ Department of Statistics University of Connecticut Storrs Connecticut USA; ^4^ Department of Orthopedics Peking University International Hospital Beijing China

**Keywords:** Allograft, Biological reconstruction, Devitalized autograft, Intercalary reconstruction, Vascularized fibular graft

## Abstract

**Objectives:**

To determine nonunion rate, fracture rate, and their risk factors following biological intercalary reconstruction for lower extremity bone tumors.

**Methods:**

A systematic review and pooled analysis were conducted. PubMed, Embase, and Wiley Cochrane Library were searched from inception up to June 01, 2020. Studies concerning biological intercalary reconstruction after resection of lower extremity bone tumors were included. Overall nonunion and fracture rates were calculated. For studies reporting patient outcomes individually with precise graft characteristics and fixation methods, the individual data were extracted. Patients with demographical and clinical characteristics, including age, sex, tumor location, graft characteristics, and fixation method, were pooled for a multivariate analysis. For each factor of interest, odds ratio (OR), 95% confidence interval (95% CI), and *p*‐value from logistic regression were reported.

**Results:**

A total of 2776 articles were identified from the initial literature search and 76 studies (2052 patients) were included. Sixty‐nine studies were case series and seven were comparative studies. The overall nonunion rate was 19% (382/2052; range: 0%–53%), and the overall fracture rate was 17% (344/2052; range: 0%–75%). Thirty of the 76 studies (362 patients) reported patients' characteristics individually and were thus included in the pooled multivariate analysis. Intramedullary nail fixation was associated with a significantly higher nonunion rate compared to plate fixation (OR = 2.2, 95% CI: 1.23–4.10, *p* = 0.009). Reconstruction with a vascularized fibula graft had a statistically non‐significant lower nonunion rate than reconstruction without the graft (OR = 0.6, 95% CI: 0.34–1.07, *p* = 0.086). Devitalized autografts had a lower fracture risk than allografts (OR = 0.3, 95% CI: 0.14–0.64, *p* = 0.002), and males tended to have higher fracture risk than females (OR = 2.1, 95% CI: 1.00–4.44, *p* = 0.049).

**Conclusions:**

Reconstruction with intramedullary nail fixation is related to an elevated risk of nonunion. Allografts and males have a higher fracture risk than devitalized autografts and females, respectively. Further high‐quality comparative analyses with large sample sizes and adequate follow‐up duration are needed to validate these findings.

## Graphical abstract

A total of 76 studies (2052 patients) were included. The overall nonunion rate was 19%, and the overall fracture rate was 17%. Thirty of the 76 studies (362 patients) reported patients' characteristics individually and were thus included in the pooled multivariate analysis. Intercalary reconstruction with intramedullary nail fixation is related to an elevated risk of nonunion. Allografts and males have a higher fracture risk than devitalized autografts and females, respectively.

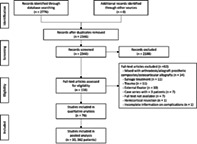

## Introduction

For bone tumors occurring in the metaphysis or diaphysis of long bones, intercalary resection followed by reconstruction is a feasible treatment option as it preserves the joint above and below the defect site.^1^ Biological reconstruction is frequently used after intercalary tumor resection. Although there is a significant risk of complications including nonunion, fracture, and infection within 3–4 years after the surgery, grafts achieve a stable state if they survive this critical period.^1^, ^2^, ^3^, ^4^, ^5^, ^6^, ^7^

Various attempts have been made to promote union and prevent fracture in intercalary reconstruction. Several types of massive bony graft are used including allografts,^2^, ^8^, ^9^ extracorporeal irradiated autografts,^10^, ^11^, ^12^ pasteurized autografts,^13^, ^14^, ^15^ and frozen autografts.^6^ These grafts can be accurately matched to conform to the configuration of a bony defect and provide initial structural strength. However, because of their avascular characteristic, the healing of massive bony grafts is generally slow,^16^ causing delayed union or nonunion and fracture in the absence of adequate fixation. Vascularized fibular grafts contain blood vessels and living osteoblasts, which accelerates union and reduces the nonunion rate.^17^ However, shape mismatch and mechanical weakness limit their independent use. Therefore, hybrid reconstruction combining massive bony grafts and free fibular grafts—known as the Capanna technique—was developed.^18^, ^19^ This method combines the advantages of mechanical strength from the massive bony graft and the capacity for bone healing and revascularization of the free fibular graft.^5^, ^20^ Various fixation devices have been used in reconstruction including plates, intramedullary nails, screws, and Kirschner wires.

There is still no well‐established optimal technique for intercalary reconstruction. Because of the generally small sample sizes of retrospective studies, nonunion rates (0%–46%)^20^, ^21^ and fracture rates (0%–45%)^12^, ^22^ reported in the literature vary considerably. Also, there is no consensus on how the rates are affected by the type of massive bony graft and implant fixation device. A more detailed analysis of published studies and reports can provide insight into this issue. To this end, in the present study, we performed a systematic review of the literature on biological intercalary reconstruction for lower extremity bone tumors, aiming at (1) determining nonunion rate and its risk factors, (2) determining fracture rate and its risk factors.

## Materials and Methods

### Search Strategy

The protocol for this review was developed in accordance with Preferred Reporting Items for Systematic Reviews and Meta‐Analyses guidelines.^23^ PubMed, Embase, and Wiley Cochrane Library were searched from inception up to June 01, 2020, using the following terms: “intercalary reconstruction,” “vascularized fibular graft,” and “Capanna technique.” We used the logical operator “OR” between search terms to increase the sensitivity of the search. The detailed search strategy was described in supplementary material.

### Inclusion and Exclusion Criteria

We included studies concerning biological intercalary reconstruction after resection of lower extremity bone tumors. The inclusion criteria are: (1) patients received intercalary resection of lower extremity bone tumors; (2) the intervention was different graft types and fixation methods used in biological intercalary reconstructions; (3) the different graft types and fixation methods were compared with each other; (4) the outcome was nonunion rate and fracture rate; (5) published clinical studies.

The exclusion criteria were as follows: (1) studies focused on endoprosthesis reconstruction, soft tissue reconstruction, or rotationplasty; (2) studies that included arthrodesis, allograft prosthetic composites, and osteoarticular allograft, where data pertaining to intercalary reconstruction could not be extracted separately; (3) studies focused on hemicortical resection/external fixator/distraction osteogenesis/induced membrane technique; (4) studies involving revision surgery; (5) studies unrelated to primary bone tumors; (6) studies mainly focused on locations other than the lower extremities; (7) studies comprising less than three cases; (8) studies lacking a description of complications from reconstruction; (9) studies with a follow‐up time <12 months; (10) animal and biomechanical studies; (11) reviews; and (12) articles not written in English.

### Study Selection

Two independent reviewers evaluated the title and abstract of all articles according to the inclusion and exclusion criteria to assess their eligibility. In cases where eligibility could not be determined based on the title and abstract, the full manuscript was reviewed. The principal investigator of the study was consulted for any disagreement between the two reviewers.

### Quality Evaluation

The methodologic quality of all included studies was analyzed by the reviewing authors based on Methodological Index for Nonrandomized Studies (MINORS) criteria.^24^

### Data Extraction

Two reviewers independently extracted data from each article using a standardized form. The following data were collected: first author, year of publication, study population characteristics, tumor type, tumor location, chemotherapy, radiation therapy, graft characteristics, fixation method, nonunion rate, and fracture rate. For studies including arthrodesis, allograft prosthetic composites, and osteoarticular allografts, only data for intercalary reconstruction were collected. For studies reporting patient outcomes individually with precise graft characteristics and fixation methods, the individual data were extracted and combined for further analysis. After independent data extractions, the reviewers compared the extracted data.

### Statistical Analysis

Statistical analysis was performed using the Statistical Package for the Social Sciences software version 16.0 (SPSS Inc., Chicago, IL) and R 3.6.2 (R Foundation for Statistical Computing, Vienna, Austria).^25^

Overall nonunion and fracture rates were calculated among the selected studies. For patients with detailed characteristics, we compared the nonunion/fracture group against the normal group for each variable. Pearson's chi‐squared test was used for nominal variables, while the Mann–Whitney test was used for continuous variables. The missing data were handled by multiple imputations using “mice” package in R.^26^

Logistic regression models were applied to assess nonunion and fracture rates.

Statistical findings, including odds ratios (ORs), corresponding 95% confidence intervals (95% CI), and *p*‐values were reported (significance was set at *p* < 0.05).

## Results

### Literature Search

**FIGURE 1:**
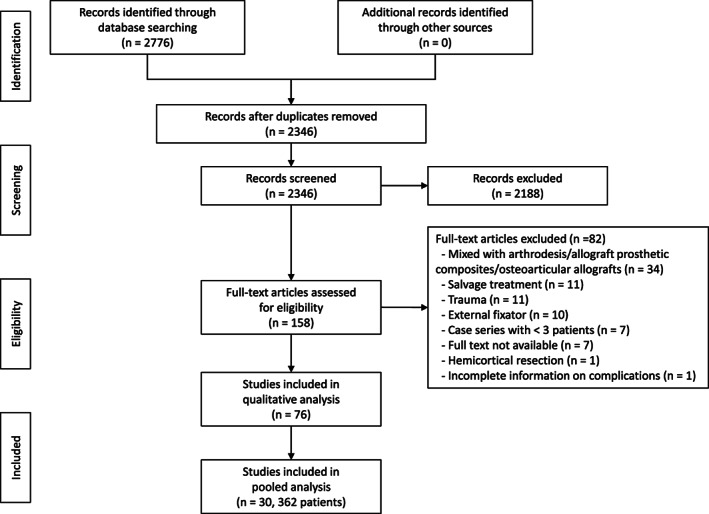
Flow diagram of the search strategy and results based on preferred reporting items for systematic reviews and meta‐analysis guidelines

A total of 2776 articles were identified from the initial literature search (Figure 1). After removing duplicate records, 2346 articles remained. Of these, 2188 were excluded based on their title and abstract. The full text of the remaining 158 articles was reviewed for eligibility; 82 were excluded, leaving 76 studies for inclusion in the review. Data of 362 patients from 30 studies individually reporting patient outcomes were pooled for analysis.

### Study Characteristics

Of the 76 studies included in the review, 69 were case series and seven were retrospective case–control studies. Two studies retrospectively compared outcomes between endoprosthetic reconstruction and intercalary allograft.^27^, ^28^ The remaining five comparative studies evaluated graft characteristics in allograft versus irradiated autograft,^11^ allograft versus pasteurized autograft,^29^ free versus pedicled vascularized fibular graft,^30^ allograft versus Capanna technique,^22^ and nonvascularized fibula graft versus irradiated autograft.^31^ The characteristics of individual studies are shown in Supplementary Table I and data from the pooled analysis are summarized in Table 1.

**TABLE 1:** Characteristics of the pooled population and results of the univariate analysis

Characteristic	Nonunion (n = 72)	Union (n = 290)	Statistics	*p* Value	Fracture (n = 46)	Non‐fracture (n = 316)	Statistics	*p* Value
Age, years*	24 (4–80)	24 (3–74)	9737	0.826	20 (3–59)	24 (4–80)	5495	0.031
Sex^†^			1.039	0.308			5.036	0.025
Male (n = 194)	43 (60)	151 (52)			30 (65)	164 (52)		
Female (n = 147)	26 (36)	121 (42)			11 (24)	136 (43)		
Unreported (n = 21)	3 (4)	18 (6)			5 (11)	16 (5)		
Duration of follow‐up, months	82 (18–300)	82 (16–313)			94 (24–313)	80 (16–300)		
Location			3.118	0.077			0.209	0.648
Femur (n = 208)	48 (67)	160 (55)			25 (54)	183 (58)		
Tibia (n = 154)	24 (33)	130 (45)			21 (46)	133 (42)		
Massive bone type^‡^			3.916	0.048			12.618	0.0004
Allograft (n = 179)	29 (40)	150 (52)			34 (74)	145 (46)		
Devitalized autograft (n = 183)	45 (63)	138 (48)			12 (26)	171 (54)		
Pasteurized autograft (n = 88)	21 (29)	67 (23)			7 (15)	81 (26)		
Irradiated autograft (n = 83)	22 (31)	61 (21)			5 (11)	78 (25)		
Frozen autograft (n = 12)	2 (3)	10 (3)			0 (0)	12 (4)		
Vascularized fibular graft			4.915	0.027			0.340	0.560
Yes (n = 198)	31 (43)	167 (58)			27 (59)	171 (54)		
No (n = 164)	41 (57)	123 (42)			19 (41)	145 (46)		
Fixation method^§^			12.238	0.0005			0.001	0.973
Plate(s) (n = 258)	41 (57)	217 (75)			30 (65)	228 (72)		
Intramedullary nail^**^ (n = 87)	29 (40)	58 (20)			10 (22)	77 (24)		
Screws or Kirschner wires (n = 17)	2 (3)	15 (5)			6 (13)	11 (3)		

*Note*: Continuous variables are reported as mean (range), and nominal variables are reported as n (%) unless otherwise indicated.

* Age was not reported for 12/362 patients; univariate analysis was based on 350 patients with a reported age.

^†^ Sex was not reported for 21/362 patients; univariate analysis was based on 341 patients with reported sex.

^‡^ Pasteurized, irradiated, and frozen autografts were combined as devitalized autograft and compared with allograft.

^§^ Univariate analysis was performed between plate(s) and intramedullary nail.

^**^ A combination of intramedullary nail and plate fixation was classified as intramedullary nail.

### Quality Assessment

The methodologic quality and risk of bias of the included studies were assessed based on MINORS scores. The mean score was 10 for case series (range: 8–11) and 15 (range: 14–17) for comparative studies; all studies were therefore deemed eligible for inclusion in the review.

### Nonunion Rate and Risk Factors

In the 76 studies included in the review, the overall nonunion rate was 19% (382/2052; range: 0%–53%). Of the 362 patients included in the multivariate analysis, nonunion occurred in 72 reconstructions (20%). In the logistic regression model (Table 2), intramedullary nail fixation showed a trend toward higher nonunion rates than plate fixation (OR = 2.2, 95% CI: 1.23–4.10, *p* = 0.009). Reconstruction with a vascularized fibula graft had a lower nonunion rate than those without a graft (OR = 0.6, 95% CI: 0.34–1.07, *p* = 0.086), but this was not statistically significant.

**TABLE 2:** Results of logistic regression of factors affecting nonunion

Variable	Odds ratio (95% CI)	*p* Value
Age*	1.0 (0.97, 1.01)	0.258
Sex^†^ (male *vs* female)	1.2 (0.69, 2.11)	0.515
Tumor location (femur *vs* tibia)	1.3 (0.72, 2.34)	0.378
Massive bone type (devitalized autograft *vs* allograft)	1.3 (0.77, 2.38)	0.301
Vascularized fibular graft (yes *vs* no)	0.6 (0.34, 1.07)	0.086
Fixation method (intramedullary nail *vs* plate)	2.2 (1.23, 4.10)	0.009^‡^

*Note*: Tumor location, massive bone type, vascularized fibular graft and fixation method were reported for all 362 patients.

* Age was not reported for 12/362 patients; logistic regression was performed after multiple imputation of missing data.

^†^ Sex was not reported for 21/362 patients; logistic regression was performed after multiple imputation of missing data.

^‡^ *p* < 0.01.

### Fracture Rate and Risk Factors

In the 76 studies included in the review, the overall fracture rate was 17% (344/2052; range: 0%–75%). Of the 362 patients included in the multivariate analysis, fracture occurred in 41 reconstructions (11%). In the logistic regression model (Table 3), devitalized autografts (OR = 0.3, 95% CI: 0.14–0.64, *p* = 0.002) had a lower fracture risk than allografts, while males (OR = 2.1, 95% CI: 1.00–4.44, *p* = 0.049) showed a trend toward higher fracture rates than females.

**TABLE 3:** Results of multivariate analysis of factors affecting fracture

Variable	Odds ratio (95% CI)	*p* Value
Age*	1.0 (0.96, 1.01)	0.163
Sex^†^ (male *vs* female)	2.1 (1.00, 4.44)	0.049^‡^
Tumor location (femur *vs* tibia)	1.2 (0.61, 2.52)	0.558
Massive bone type (devitalized autograft *vs* allograft)	0.3 (0.14, 0.64)	0.002^**^
Vascularized fibular graft (yes *vs* no)	1.0 (0.49, 2.12)	0.966
Fixation method (intramedullary nail *vs* plate)	1.3 (0.56, 3.24)	0.506

*Note*: Tumor location, massive bone type, vascularized fibular graft and fixation method were reported for all 362 patients.

* Age was not reported for 12/362 patients; logistic regression was performed after multiple imputation of missing data.

^†^ Sex was not reported for 21/362 patients; logistic regression was performed after multiple imputation of missing data.

^‡^ *p* < 0.05.

^**^ *p* < 0.01.

## Discussion

Intercalary reconstruction is widely used following resection of primary bone tumors in the extremities. Here we have provided the first comprehensive analysis of aggregate data on nonunion and fracture rates following biological intercalary reconstruction and identified factors influencing these rates.

### Nonunion Rate and Risk Factors

The mean nonunion rate was 19%, with considerable heterogeneity across studies (0%–53%). The most important risk factor for nonunion was reconstruction with intramedullary nail fixation. It is thought that intramedullary nails do not provide adequate rotational stability in intercalary reconstruction even when interlocking screws are applied.^32^ Fixation using only intramedullary nails was shown to be associated with a higher risk of nonunion and bridging osteosynthesis with plate fixation has been recommended instead.^9^, ^21^, ^33^, ^34^ Some studies favored intramedullary interlocking nails in combination with small plates to provide better rotational stability.^11^, ^35^ This hybrid fixation (n = 21) did not show superiority over intramedullary nail only fixation in nonunion rate in our multivariate analysis (OR = 1.08, *p* = 0.893).

It is generally accepted that the viability of vascularized fibula graft is superior to allograft, which promotes bone healing through its intrinsic blood supply and living cells.^36^, ^37^ A comparative study with a small sample size (29 patients) reported that vascularized fibula grafts reduced the risk of allograft revision although there were no statistically significant differences in union rate or time to union between reconstruction with or without such a graft.^22^ Several authors have previously recommended using vascularized fibula grafts in combination with allografts or devitalized autografts to reduce nonunion rate.^7^, ^21^, ^38^ In our study, a lesser proportion of reconstructions had nonunion with a vascularized fibula graft than without one (OR = 0.6, 95% CI: 0.34–1.07, *p* = 0.086), although this failed to reach statistical significance.

Massive bone type and tumor location have been described as associated with nonunion rate. It was considered that the irradiated autograft had a lower rate of nonunion than allograft^11^ and the pasteurized autograft has a higher rate.^29^ Femoral reconstruction^33^ and advanced age in patients^21^, ^33^ were shown to be related to a higher risk of nonunion; however, we did not observe an association between these factors and nonunion rate.

### Fracture Rate and Risk Factors

The overall fracture rate was 17% and varied considerably across studies (0%–75%). Devitalized autografts had a lower fracture risk than allografts. The revascularization of the allograft begins after reconstruction. Living cells from the host body populate on the outer surface of the allograft, and small erosive cavities are formed through osteoclastic resorption.^39^ However, this remodeling process can reduce the structural integrity of the allograft, which increases the rate of fracture.^22^, ^40^ Contrary to our findings, irradiated^11^ and pasteurized^29^ autografts had a higher fracture rate than allografts in comparative studies, although the difference was nonsignificant. We attribute the discrepancy to small sample sizes in these two studies, in which the number of fractures differed by only one case between the two groups, with fewer than 20 patients per group.

Males was associated with a higher fracture risk than females. A previous study suggested that children and adolescents were at a higher risk of fracture because they are likely to place high functional demands on the graft.^40^ Similarly, we infer that male patients are more susceptible to fractures than females because of a higher level of physical activity.

The effect of fixation method on fracture risk of intercalary reconstruction has been widely discussed. Early studies reported that plate fixation had a higher rate of fracture than intramedullary nails.^41^, ^42^, ^43^ The observation that the fracture line frequently involves screw holes suggests that boring into cortical bone creates stress‐concentrating defects that are vulnerable to fracture. Therefore, intramedullary fixation has been advocated to avoid cortical penetration. However, the application of intramedullary nails is restricted to tumors close to the articular surface. Further findings indicated that fractures frequently appeared in the areas where the allograft was not covered by the internal fixation, which could have been reduced had the entire graft been protected *via* extracortical support.^9^, ^42^, ^44^ As this concept has gained acceptance, a bridging plate spanning two host‐graft junctions instead of two small plates has been used and was shown to reduce fracture rates.^5^, ^19^, ^29^, ^33^, ^44^ Multiple‐plate fixation using a bridging plate results in a more stable fixation.^30^, ^45^, ^46^ The construct may consist of two long plates or a long plate on one side and two shorter plates on the opposite side. However, the effectiveness of this fixation method requires further investigation. We found no significant difference in fracture rate between plate and intramedullary nail fixation. There was considerable heterogeneity within the plate fixation group, and we were unable to perform a subgroup analysis because many studies did not clearly describe the plate placement method.

### Limitations

The present study had several limitations. Firstly, there was variability across the included studies in terms of study design, operative technique used, and reporting of clinical outcomes, which precluded a formal meta‐analysis. To make the best use of available data to identify factors affecting bone healing and fracture, we carried out a pooled analysis using patient data collected individually. The 362 patients included in the pooled analysis can be regarded as a sample of the 2052 patients. The nonunion rate of the sample (20%) was close to the whole (19%). However, the difference in fracture rates (sample *vs* whole, 11% *vs* 17%) might weaken the validity of analysis. Because we were limited by the incomplete reporting of case data, we mainly focused on variables related to the surgical technique, including grafted massive bone type, use of vascularized fibular grafts, and fixation method. However, other factors can impact clinical outcomes such as location of osteotomy,^9^, ^19^, ^21^, ^44^, ^47^ chemotherapy,^2^, ^9^, ^19^, ^20^, ^21^, ^47^ and the use of intramedullary cement.^2^, ^48^ These were not considered in the pooled analysis and are potential confounds.

The definition of nonunion is inconsistent across studies; in most cases, union is defined radiologically (bone bridging at least three of the four cortices in biplanar X‐rays), while some studies^14^, ^49^, ^50^ have included a clinical requirement (absence of pain on full weight‐bearing). Nonunion is regarded as a failure of union 1 year after operation^2^, ^11^, ^21^, ^40^; however, some studies defined nonunion as no progression of union at 6 months after operation^29^, ^46^ or when surgical intervention is needed to facilitate union.^33^, ^34^, ^38^ Because of the variability in follow‐up times (mean, 82 months; range: 16–313 months) across included studies, fractures may be underreported because they were overlooked in studies with a shorter follow‐up duration. These discrepancies between studies could undermine the validity of comparisons of nonunion rates and fracture rates.

### Conclusion

Our systematic review has demonstrated that the overall nonunion rate and fracture rate after biological intercalary reconstruction are 19% and 17%, respectively. Reconstruction with intramedullary nail fixation has a higher risk of nonunion. Allografts and males are also associated with an increased risk of fracture compared to devitalized autografts and female sex, respectively. The superiority of devitalized autografts over allografts in terms of fracture rate has not been previously reported. Although further high‐quality comparative studies with large sample sizes and adequate follow‐up times are needed to confirm our results, our findings can guide surgeons in the selection of the appropriate technique for intercalary reconstruction.

## Author Contributions

Conception and design of study: Tao Ji, Wei Guo.

Acquisition of data: Siyi Huang, Zhili Xing, Tao Ji.

Analysis and/or interpretation of data: Siyi Huang, Hongfei Li, Tao Ji.

Drafting the manuscript: Siyi Huang, Tao Ji.

Revising the manuscript critically for important intellectual content: Tao Ji, Wei Guo.

## Funding Information

This work was supported by the Peking University People's Hospital Scientific Research Development Funds (RDL2020‐01); the National Natural Science Foundation of China (81872180); and The Capital Health Research and Development of Special (2018‐2‐4088).

## Conflict of Interest

The authors declare that the research was conducted in the absence of any commercial or financial relationships that could be construed as a potential conflict of interest.

## Ethics Statement

All authors listed above meet the authorship criteria according to the latest guidelines of the International Committee of Medical Journal Editors. All authors are in agreement with the manuscript.

## Disclosure Statement

All authors have nothing to disclose.

## Supporting information


**Appendix S1**. Supporting Information

**Appendix S2**. Supporting Information
