# Supplementary material for: Factors Influencing Nonunion and Fracture Following Biological Intercalary Reconstruction for Lower‐Extremity Bone Tumors: A Systematic Review and Pooled Analysis
Source: Orthop Surg. 2022 Oct 20;14(12):3261–7. doi: 10.1111/os.13546 (PMC9732628; doi:10.1111/os.13546)
Supplement: Supplementary file 1 — Appendix S1. Supporting Information [file OS-14-3261-s001.docx]

**Search strategy**

The query formulation, ((intercalary reconstruction) OR (vascularized fibular graft)) OR (Capanna technique), was used in all three database, PubMed, Embase, and Wiley Cochrane Library.

**PubMed:**

Pubmed can automatically match search terms to synonyms and MeSH terms. The search details are displayed below.

reconstruction: *"reconstruct"[All Fields] OR "reconstructability"[All Fields] OR "reconstructable"[All Fields] OR "reconstructed"[All Fields] OR "reconstructible"[All Fields] OR "reconstructing"[All Fields] OR "reconstructional"[All Fields] OR "reconstructive surgical procedures"[MeSH Terms] OR ("reconstructive"[All Fields] AND "surgical"[All Fields] AND "procedures"[All Fields]) OR "reconstructive surgical procedures"[All Fields] OR "reconstruction"[All Fields] OR "reconstructions"[All Fields] OR "reconstructive"[All Fields] OR "reconstructs"[All Fields]*

vascularized: *"blood vessels"[MeSH Terms] OR ("blood"[All Fields] AND "vessels"[All Fields]) OR "blood vessels"[All Fields] OR "vascular"[All Fields] OR "neovascularization, pathologic"[MeSH Terms] OR ("neovascularization"[All Fields] AND "pathologic"[All Fields]) OR "pathologic neovascularization"[All Fields] OR "vascularisation"[All Fields] OR "vascularization"[All Fields] OR "vascularisations"[All Fields] OR "vascularise"[All Fields] OR "vascularised"[All Fields] OR "vascularities"[All Fields] OR "vascularitis"[All Fields] OR "vascularity"[All Fields] OR "vascularizations"[All Fields] OR "vascularize"[All Fields] OR "vascularized"[All Fields] OR "vascularizes"[All Fields] OR "vascularizing"[All Fields] OR "vasculars"[All Fields]*

fibular: *"fibula"[MeSH Terms] OR "fibula"[All Fields] OR "fibular"[All Fields] OR "fibulare"[All Fields]*

graft: *"graft's"[All Fields] OR "grafted"[All Fields] OR "graftings"[All Fields] OR "transplantation"[Subheading] OR "transplantation"[All Fields] OR "grafting"[All Fields] OR "transplantation"[MeSH Terms] OR "grafts"[All Fields] OR "transplants"[MeSH Terms] OR "transplants"[All Fields] OR "graft"[All Fields]*

technique: *"methods"[Subheading] OR "methods"[All Fields] OR "techniques"[All Fields] OR "methods"[MeSH Terms] OR "technique"[All Fields] OR "technique's"[All Fields]*

**Embase:**

The search details are displayed below.

*'intercalary reconstruction' OR (intercalary AND ('reconstruction'/exp OR reconstruction)) OR 'vascularized fibular graft'/exp OR 'vascularized fibular graft' OR (vascularized AND fibular AND ('graft'/exp OR graft)) OR 'capanna technique' OR (capanna AND ('technique'/exp OR technique))*

**Wiley Cochrane Library:**

In Wiley Cochrane Library, the website notes that word variations have been searched, but no search details can be found.
