# Supplementary material for: Factors Influencing Nonunion and Fracture Following Biological Intercalary Reconstruction for Lower‐Extremity Bone Tumors: A Systematic Review and Pooled Analysis
Source: Orthop Surg. 2022 Oct 20;14(12):3261–7. doi: 10.1111/os.13546 (PMC9732628; doi:10.1111/os.13546)
Supplement: Supplementary file 2 — Appendix S2. Supporting Information [file OS-14-3261-s002.docx]

Supplementary Table I. Included study characteristics and results summary

| Study | Patient number | Average age (years, range) | Male:female | Followup (months, range) | Tumor type† | Tumor location | Chemotherapy | Radiation therapy | Graft characteristic | Fixation method | Nonunion | Fracture | Part of the study | Included in quantitative analysis | MINORS score |
| --- | --- | --- | --- | --- | --- | --- | --- | --- | --- | --- | --- | --- | --- | --- | --- |
| Brien, et al. (1994) ^1^ | 9 | 26, (12-63) | 4:5 | 37, 17-84 | OS, Ewing, ada | tibia | 4 (44%) | 0 (0%) | allograft | plate, IMN, screw | 2 (22%) | 1 (11%) | Yes | Yes | 9 |
| Alman, et al. (1995) ^2^ | 6 | 11, (5-15) | NR | 55, 44-75 | OS | femur, tibia | 6 (100%) | 0 (0%) | allograft | plate | 1 (17%) | 4 (67%) | Yes | Yes | 10 |
| Voggenreiter, et al. (1995) ^3^ | 10 | 41, 22-62 | 7:3 | 48, 12-108 | ada, synovial sacroma, ABC, etal | femur, tibia | 2 (20%) | 4 (40%) | allograft | plate, IMN | 4 (40%) | 1 (10%) | Yes | Yes | 9 |
| Hsu, et al. (1997) ^4^ | 3 | 36, 22-62 | 0:3 | 36, 24-60 | cho, ada, soft tissue sacroma | femur, tibia | NR | NR | VFG | plate | 0 (0%) | 0 (0%) | Yes | No | 8 |
| Ortiz-Cruz, et al. (1997) ^5^ | 100 | 28, 4-69 | 47:53 | 73, 24-220 | Ewing, cho, ada, etal | femur, tibia, humerus, radius, fibula, ulna | NR | NR | allograft | plate, IMN, screw | 31 (30%) | 18 (17%) | No | No | 10 |
| Ozaki, et al. (1997) ^6^ | 12 | 18, 7-54 | 7:5 | 30, 24-42 | Ewing, OS, ada, MFH | tibia | 7 (58%) | 5 (42%) | allograft+VFG | plate, screw, Kirschner wires | 1 (8%) | 4 (33%) | No | Yes | 9 |
| Araki, et al. (1999) ^7^ | 6 | 37, 16-80 | 3:3 | 71, 52-100 | OS, angiosarcoma, MFH | femur, tibia | NR | NR | irradiated autograft | plate, IMN | 3 (50%) | 1 (17%) | Yes | Yes | 10 |
| Ceruso, et al. (2001) ^8^ | 52 | 19, 2-65 | NR | 46, 3.5-114 | OS, Ewing, ada, etal | femur, tibia, humerus | NR | NR | allograft+VFG | plate, screw, Kirschner wires | 2 (4%) | 11 (21%) | No | No | 9 |
| Gerrand, et al. (2003) ^9^ | 8 | 38, 15-65 | 4:4 | 54, 16-97 | OS, Ada, Ewing, etal | femur, tibia | 3 (38%) | 2 (25%) | allograft | plate | 1 (13%) | 0 (0%) | Yes | Yes | 9 |
| Muscolo, et al. (2004) ^10^ | 59 | 28, 4-66 | 24:35 | 60, 24-268 | OS, cho, Ewing, etal | femur, tibia | 35 (59%) | NR | allograft | plate, IMN, screw | 9 (15%) | 4 (7%) | No | No | 11 |
| Muscolo, et al. (2004) ^11^ | 13 | 18, 9-40 | 5:8 | 63, 24-144 | OS | femur, tibia | 13 (100%) | NR | allograft | plate, IMN, screw | 2 (15%) | 3 (23%) | No | No | 10 |
| Sakayama, et al. (2004) ^12^ | 4 | 30, 21-40 | 2:2 | 73, 64-81 | OS, cho, MFH | femur, tibia | 0 (0%) | NR | pasteurized autograft | IMN, screw | 0 (0%) | 0 (0%) | Yes | Yes | 9 |
| Chang, et al. (2005) ^13^ | 12 | 25, 6-71 | NR | 24 | NR | femur, tibia, humerus | NR | NR | allograft+VFG | NR | 1 (8%) | 0 (0%) | No | No | 9 |
| Chen, et al. (2005) ^14^ | 26 | 25, 9-74 | 16:10 | 70, 24-130 | OS, MFH, cho, etal | femur, tibia | 25 (96%) | NR | allograft, irradiated autograft | plate, IMN | 7 (27%) | 4 (15%) | Yes | Yes | 15# |
| Deijkers, et al. (2005) ^15^ | 35 | 24*, 11-68 | 15:20 | 86 | OS, cho, ada, etal | femur, tibia | 18 (51%) | NR | allograft, VFG | plate, IMN, staple | 9 (26%) | 12 (34%) | No | No | 11 |
| Pollock, et al. (2005) ^16^ | 7 | 23, 11-37 | 2:4 | NR | OS, cho, Ewing, etal | femur, tibia | NR | NR | allograft+VFG, irradiated autograft+VFG | plate, IMN | 2 (29%) | 1 (14%) | Yes | Yes | 9 |
| Moran, et al. (2006) ^17^ | 7 | 11 | 5:2 | 52 | Ewing, OS, cho | femur, tibia | 4 (57%) | NR | allograft+VFG | plate, screw | 2 (29%) | 2 (29%) | No | No | 9 |
| Muramatsu, et al. (2006) ^18^ | 6 | 31, 5-55 | 6:0 | 39, 12-72 | OS, GCT, MFH | femur | 2 (33%) | NR | VFG | plate, IMN | 1 (17%) | 0 (0%) | No | No | 9 |
| Capanna, et al. (2007) ^19^ | 90 | NR | NR | 108, 26-204 | NR | femur, tibia | NR | NR | allograft+VFG | plate, screw | 8 (9%) | 12 (13%) | No | No | 10 |
| Jeon, et al. (2007) ^20^ | 21 | 35, 11-59 | 16:5 | 74, 28-181 | OS, MFH, Ewing, etal | femur, tibia | 18 (86%) | NR | pasteurized autograft | plate, IMN | 5 (24%) | 2 (10%) | No | Yes | 10 |
| Krieg, et al. (2007) ^21^ | 16 | 17, 8-55 | 9:7 | 50, 24-96 | NR | femur | 14 (88%) | NR | irradiated autograft (+VFG) | plate, IMN, screw | 3 (16%) | 1 (6%) | No | No | 11 |
| Sugiura, et al. (2007) ^22^ | 15 | 26, 10-56 | 7:8 | 48, 24-108 | OS, cho, ada, etal | femur, tibia | NR | NR | pasteurized autograft+VFG | plate, IMN | 3 (20%) | 2 (13%) | No | No | 10 |
| Muscolo, et al. (2008) ^23^ | 12 | 8, 2-10 | 8:4 | 52, 24-124 | OS, Ewing | femur, tibia | NR | NR | allograft | plate, IMN | 2 (17%) | 3 (25%) | Yes | No | 11 |
| Abed, et al. (2009) ^24^ | 25 | 20, 5-52 | 16:9 | 140, 28-213 | OS, Ewing, ada, etal | femur, tibia | 18 (72%) | NR | allograft+VFG | plate, screw , Kirschner wires | 1 (4%) | 9 (36%) | No | No | 10 |
| Innocenti, et al. (2009) ^25^ | 21 | 18, 5-52 | 13:8 | 139, 28-204 | OS, Ewing, ada, etal | tibia | 15 (71%) | NR | allograft+VFG | plate, screw , Kirschner wires | 1 (5%) | 5 (24%) | No | Yes | 10 |
| Wang, et al. (2009) ^26^ | 33 | 12, 8-16 | 21:12 | 38, 12-72 | OS, Ewing, cho, etal | femur, tibia | 33 (100%) | NR | allograft | IMN+screw | 2 (6%) | 1 (3%) | No | No | 8 |
| Zimel, et al. (2009) ^27^ | 38 | 16, 4-45 | 20:18 | NR | OS, Ewing , cho, etal | femur | 34 (89%) | 2 (5%) | allograft | NR | 6 (16%) | 1 (3%) | Yes | No | 14# |
| Hariri, et al. (2010) ^28^ | 38 | 12, 4-23 | 23:15 | 91, 5-221 | OS, Ewing sarcoma, synovial sarcoma, etal | femur, tibia | 37 (97%) | 1 (3%) | VFG | plate | 4 (11%) | 8 (21%) | No | No | 11 |
| Jager, et al. (2010) ^29^ | 4 | 12, 8-15 | 3:1 | 46, 37-63 | ABC, OS, Ewing | femur, tibia | NR | NR | allograft+VFG | plate | 1 (25%) | 1 (25%) | Yes | Yes | 9 |
| Li, et al. (2010) ^30^ | 11 | 19, 11-32 | 5:6 | 34, 17-53 | OS, cho, ada, etal | femur, tibia | 8 (73%) | 1 (9%) | allograft+VFG | plate | 1 (9%) | 0 (0%) | No | Yes | 10 |
| Yang, et al. (2010) ^31^ | 17 | 18, 6-34 | 10:7 | NR | GCT, OS, synovial sarcoma, etal | femur, tibia | NR | NR | allograft+VFG | plate | 0 (0%) | 0 (0%) | No | No | 9 |
| Brunet, et al. (2011) ^32^ | 13 | 22, 14-50 | 6:7 | 48* | OS, Ewing, ada | femur, tibia | 10 (77%) | 2 (15%) | allograft (+VFG) | plate, IMN | 4 (31%) | 2 (15%) | No | Yes | 9 |
| Li, et al. (2011) ^33^ | 8 | 17, 11-28 | 3:5 | 38, 7-58 | OS, Ewing, ada | tibia | 6 (75%) | NR | allograft+VFG | plate | 2 (25%) | 0 (0%) | No | No | 9 |
| Puri, et al. (2011) ^34^ | 32 | 12, 2-16 | 25:17 | 42, 12-86 | OS, Ewing | femur, tibia | NR | NR | allograft (+VFG), irradiated autograft | NR | 2 (6%) | 4 (13%) | Yes | No | 8 |
| Aponte-Tinao, et al. (2012) ^35^ | 83 | 26, 2-80 | 48:35 | 61*, 24-182 | OS, Ewing, cho, etal | femur | 53 (64%) | NR | allograft | plate, screw, IMN | 20 (24%) | 14 (17%) | No | No | 10 |
| Farfalli, et al. (2012) ^36^ | 26 | 25, 4-57 | 13:13 | 73, 9-176 | OS, cho, Ewing, etal | tibia | 16 (62%) | 0 (0%) | allograft | plate, IMN | 2 (8%) | 3 (12%) | No | Yes | 11 |
| Frisoni, et al. (2012) ^37^ | 101 | 20, 4-74 | 52:49 | 108, 24-238 | OS, Ewing, spindle cell sarcoma, etal | femur | 80 (79%) | 0 (0%) | allograft (+VFG) | plate, IMN, screw | 53 (46%) | 31 (27%) | No | No | 11 |
| Mottard, et al. (2012) ^38^ | 15 | 14, 7-25 | 11:4 | 57, 22-99 | OS, cho, ada | tibia | 12 (80%) | NR | irradiated autograft+VFG | plate | 4 (27%) | 0 (0%) | No | Yes | 9 |
| Sugiura, et al. (2012) ^39^ | 19 | 27, 10-58 | 10:9 | 103, 29-208 | OS, cho, ada, etal | femur, tibia | 13 (68%) | 1 (5%) | pasteurized autograft (+VFG) | NR | 6 (32%) | 2 (11%) | Yes | Yes | 10 |
| Tanaka, et al. (2012) ^40^ | 9 | 16, 11-20 | 4:5 | 100, 23-248 | OS, ada, cho | femur, tibia | 8 (89%) | 0 (0%) | VFG, pasteurized autograft+VFG, irradiated autograft+VFG | plate, kirschner wire | 2 (22%) | 1 (11%) | Yes | Yes | 9 |
| Nakamura, et al. (2013) ^41^ | 6 | 10, 4-23 | 3:3 | 41, 16-79 | Ewing | femur | 6 (100%) | NR | irradiated autograft | plate | 2 (33%) | 0 (0%) | No | Yes | 10 |
| Niethard, et al. (2013) ^42^ | 11 | 14, 4-43 | 6:5 | 69, 12-144 | Ewing, ada, OS, etal | femur, tibia | 11 (100%) | 3 (27%) | VFG | plate | 3 (27%) | 1 (9%) | No | No | 10 |
| Rabitsch, et al. (2013) ^43^ | 12 | 18, 11-31 | 4:8 | 39, 2-88 | Ewing, OS, liposarcoma, etal | femur, tibia | 7 (58%) | 2 (17%) | allograft+VFG | NR | 4 (33%) | 4 (33%) | No | No | 9 |
| Umer, et al. (2013) ^44^ | 31 | 12, 6-16 | 18:13 | NR | OS, Ewing, cho, etal | femur, tibia | NR | NR | autoclaved autograft+VFG, autoclaved autograft+NVFG | plate, screw, IMN | 1 (3%) | 2 (6%) | Yes | No | 9 |
| Bus, et al. (2014) ^45^ | 78 | NR | 40:38 | NR | OS, ada, Ewing, etal | femur, tibia, humerus, radius | 45 (58%) | 7 (9%) | allograft | plate, IMN, screw | 29 (37%) | 23 (29%) | Yes | No | 11 |
| Han, et al. (2014) ^46^ | 30 | 25 | 12:18 | 79, 14-150 | OS, ada, Ewing, etal | femur, tibia, humerus, fibula | 22 (73%) | 0 (0%) | allograft, pasteurized autograft | plate, IMN, screw | 16 (53%) | 5 (17%) | No | No | 14# |
| Igarashi, et al. (2014) ^47^ | 6 | 15, 13-60 | 3:3 | NR | OS, cho, leiomyosacroma | femur, tibia | NR | NR | frozen autograft | NR | 0 (0%) | 1 (17%) | Yes | No | 9 |
| Shin, et al. (2014) ^48^ | 4 | 35, 10-61 | 2:2 | 25, 18-32 | OS, Myxofibrosarcoma | tibia | 4 (100%) | 1 (25%) | allograft | plate | 0 (0%) | 0 (0%) | Yes | No | 9 |
| Aponte-Tinao, et al. (2015) ^49^ | 35 | 17, 2-50 | 19:16 | 108, 12-276 | OS | femur, tibia | 35 (100%) | NR | allograft | NR | 3 (9%) | 11 (31%) | No | No | 10 |
| Erol, et al. (2015) ^50^ | 10 | 11, 4-18 | 6:4 | 44, 33-73 | Ewing, OS | femur, tibia | 10 (100%) | 2 (20%) | VFG, allograft+VFG | plate | 0 (0%) | 1 (10%) | Yes | Yes | 10 |
| Ogura, et al. (2015) ^51^ | 11 | 29, 11-63 | 7:4 | 68, 25-131 | OS, cho, Ewing, etal | femur, tibia | 8 (73%) | 1 (9%) | pasteurized autograft+VFG, frozen autograft+VFG | plate, screw | 0 (0%) | 1 (9%) | No | Yes | 11 |
| Qu, et al. (2015) ^52^ | 27 | 28, 9-73 | 16:11 | 63, 15-116 | OS, Ewing, cho, etal | femur, tibia, humerus, radius, ulna | 18 (67%) | 1 (4%) | pasteurized autograft | plate, IMN | 4 (15%) | 1 (4%) | No | No | 9 |
| Weichman, et al. (2015) ^53^ | 12 | 16, 3-49 | 5:7 | 41, 21-104 | OS, Ewing, ada | femur, tibia | 10 (83%) | 5 (42%) | allograft+VFG | plate | 3 (25%) | 3 (25%) | No | Yes | 10 |
| Houdek, et al. (2016) ^54^ | 18 | 11, 5-18 | 9:9 | 96, 24-180 | OS, Ewing, ada, etal | femur, tibia | NR | NR | allograft+VFG | plate | 6 (33%) | 7 (39%) | No | No | 10 |
| Li, et al. (2016) ^55^ | 8 | 16, 10-26 | 3:5 | 49, 37-71 | OS, Ewing, cho | femur | 6 (75%) | 1 (13%) | frozen autograft+VFG | plate, screw | 0 (0%) | 1 (13%) | No | No | 10 |
| Emori, et al. (2017) ^56^ | 12 | NR | NR | NR | NR | femur, tibia, forearm, humerus | NR | NR | VFG, irradiated autograft+VFG | plate, IMN, screw | 1 (8%) | 9 (75%) | Yes | No | 9 |
| Gupta, et al. (2017) ^57^ | 46 | 33, 14-77 | 26:20 | 92*, 4-288 | OS, cho, Ewing, etal | femur, tibia, humerus | 22 (48%) | 3 (7%) | allograft | plate | 3 (7%) | 2 (4%) | No | No | 10 |
| Manfrini, et al. (2017) ^58^ | 47 | 14, 6-38 | 33:14 | 84*, 7-231 | OS, Ewing, ada | tibia | 40 (85%) | 1 (2%) | allograft+VFG | plate, screw | 1 (2%) | 8 (17%) | No | No | 15# |
| Zekry, et al. (2017) ^59^ | 7 | 18, 6-60 | 4:3 | 75, 28-164 | OS | femur, tibia | 6 (86%) | NR | frozen autograft | plate, IMN | 2 (29%) | 0 (0%) | Yes | Yes | 10 |
| Aponte-Tinao, et al. (2018) ^60^ | 13 | 7, 2-10 | 9:4 | 158, 120-220 | OS, Ewing | femur, tibia | NR | NR | allograft | NR | 0 (0%) | 3 (23%) | Yes | No | 10 |
| Campanacci, et al. (2018) ^61^ | 23 | 16, 5-40 | 15:8 | 141, 24-313 | OS, cho, Ewing, etal | femur | 19 (83%) | 0 (0%) | allograft+VFG | plate, screw | 2 (9%) | 6 (26%) | No | Yes | 10 |
| Houdek ^62^, et al. (2018) | 29 | 12, 5-16 | 15:14 | 156, 36-288 | OS, Ewing | femur, tibia | 22 (76%) | NR | allograft (+VFG) | plate, IMN | 10 (34%) | 13 (45%) | No | No | 17# |
| Ikuta, et al. (2018) ^63^ | 19 | 22, 11-61 | 13:6 | 91, 28-208 | OS, leiomyosarcoma, rhabdomyosarcoma | femur, tibia | 18 (95%) | 0 (0%) | pasteurized autograft (+VFG) | plate, IMN | 10 (53%) | 1 (5%) | Yes | Yes | 10 |
| Lee ^64^, et al. (2018) | 71 | NR | NR | NR | NR | femur, tibia, humerus, plevis | NR | NR | pasteurized autograft | plate, IMN, screw | 8 (11%) | 2 (3%) | Yes | No | 9 |
| Puri, et al. (2018) ^65^ | 70 | 17, 1-36 | 53:17 | 56, 1-127 | OS, Ewing, ada | femur, tibia, humerus, ulna | 70 (100%) | 0 (0%) | irradiated autograft | plate, IMN | 20 (29%) | 4 (6%) | No | No | 10 |
| Ghoneimy, et al. (2019) ^66^ | 41 | 10, 5-17 | 24:17 | 49, 12-104 | Ewing, OS | femur | 40 (98%) | 1 (2%) | VFG | plate | 5 (12%) | 12 (29%) | No | No | 9 |
| Krieg, et al. (2019) ^67^ | 8 | 28, 11-59 | 4:4 | 77, 60-108 | Ewing, ada, OS | Tibia | 4 (50%) | 1 (13%) | irradiated autograft+VFG | plate | 0 (0%) | 1 (13%) | No | Yes | 10 |
| Li, et al. (2019) ^68^ | 60 | 21, 5-54 | 35:25 | 52, 12-135 | OS, Ewing, cho, etal | femur, tibia | 37 (62%) | NR | allograft+VFG | plate | 0 (0%) | 1 (2%) | No | No | 9 |
| Liu, et al. (2019) ^69^ | 15 | 22, 10-40 | 9:6 | 65, 31-131 | OS | femur | 15 (100%) | NR | pasteurized autograft+VFG | plate | 0 (0%) | 1 (7%) | No | Yes | 10 |
| Oike, et al. (2019) ^70^ | 14 | 35, 11-58 | 6:8 | 188, 123-292 | cho, OS, Ewing | femur, tibia | 6 (43%) | 0 (0%) | irradiated autograft (+VFG), irradiated autograft+NVFG | plate, IMN | 5 (36%) | 0 (0%) | Yes | Yes | 10 |
| Ruiz-Moya, et al. (2019) ^71^ | 17 | 9, 2-13 | 9:8 | 41, 18-79 | Ewing, OS | femur, tibia | NR | NR | allograft+VFG, VFG | NR | 3 (24%) | 9 (47%) | Yes | No | 10 |
| Salunke, et al. (2019) ^72^ | 28 | 20, 9-45 | NR | 24, 12-57 | Ewing, OS | femur | 28 (100%) | 7 (25%) | NVFG, irradiated autograft | plate | 3 (11%) | 1 (4%) | No | Yes | 14# |
| Shemesh, et al. (2019) ^73^ | 3 | 49, 41-66 | NR | 136, 132-144 | cho | femur | NR | 0 (0%) | allograft | plate, IMN | 1 (33%) | 0 (0%) | Yes | Yes | 10 |
| Albergo, et al. (2020) ^74^ | 71 | 16*, 6-55 | 45:26 | 129*, 12-311 | OS, Ewing, cho, etal | femur | 63 (89%) | NR | allograft | NR | 4 (6%) | 17 (24%) | Yes | No | 16# |
| Mihara, et al. (2020) ^75^ | 6 | 38, 16-63 | 4:2 | 71, 16-138 | OS, cho, ada, etal | femur, tibia | NR | NR | irradiated autograft+VFG | plate, IMN | 2 (33%) | 0 (0%) | Yes | No | 9 |
| Sanders, et al. (2020) ^76^ | 131 | 19*, 2-71 | 68:63 | 168* | OS, Ewing, cho, etal | femur, tibia | 92 (70%) | 5 (4%) | allograft | plate, IMN | 21 (16%) | 25 (19%) | No | No | 11 |

MINORS = Methodological Index for Non-Randomized Studies; NR = not reported; IMN = intramedullary nail; VFG = vascularized fibular graft; NVFG = nonvascularized fibular graft; OS = osteosarcoma; cho = chondrosarcoma; Ewing = Ewing sarcoma; ada = adamantinoma; ABC = aneurysmal bone cyst; MFH = malignant fibrous histiocytoma; GCT = giant cell tumor; *, median number; #, comparative study; †, the three most common types of tumors were listed in descending order.

1. Brien EW, Terek RM, Healey JH, Lane JM. Allograft reconstruction after proximal tibial resection for bone tumors. An analysis of function and outcome comparing allograft and prosthetic reconstructions. Clinical orthopaedics and related research. 1994(303):116-27.

2. Alman BA, De Bari A, Krajbich JI. Massive allografts in the treatment of osteosarcoma and Ewing sarcoma in children and adolescents. J Bone Joint Surg Am. 1995;77(1):54-64.

3. Voggenreiter G, Klaes W, Assenmacher S, Schmit-Neuerburg KP. Massive intercalary bone allografts in the treatment of primary and secondary bone tumors. A report on 21 cases. Arch Orthop Trauma Surg. 1995;114(6):308-18.

4. Hsu RW, Wood MB, Sim FH, Chao EY. Free vascularised fibular grafting for reconstruction after tumour resection. J Bone Joint Surg Br. 1997;79(1):36-42.

5. Ortiz-Cruz E, Gebhardt MC, Jennings LC, Springfield DS, Mankin HJ. The results of transplantation of intercalary allografts after resection of tumors. A long-term follow-up study. J Bone Joint Surg Am. 1997;79(1):97-106.

6. Ozaki T, Hillmann A, Wuisman P, Winkelmann W. Reconstruction of tibia by ipsilateral vascularized fibula and allograft. 12 cases with malignant bone tumors. Acta Orthop Scand. 1997;68(3):298-301.

7. Araki N, Myoui A, Kuratsu S, et al. Intraoperative extracorporeal autogenous irradiated bone grafts in tumor surgery. Clin Orthop Relat Res. 1999(368):196-206.

8. Ceruso M, Falcone C, Innocenti M, Delcroix L, Capanna R, Manfrini M. Skeletal reconstruction with a free vascularized fibula graft associated to bone allograft after resection of malignant bone tumor of limbs. Handchir Mikr. 2001;33(4):277-82.

9. Gerrand CH, Griffin AM, Davis AM, Gross AE, Bell RS, Wunder JS. Large segment allograft survival is improved with intramedullary cement. J Surg Oncol. 2003;84(4):198-208.

10. Muscolo DL, Ayerza MA, Aponte-Tinao L, Ranalletta M, Abalo E. Intercalary femur and tibia segmental allografts provide an acceptable alternative in reconstructing tumor resections. Clinical orthopaedics and related research. 2004(426):97-102.

11. Muscolo DL, Ayerza MA, Aponte-Tinao LA, Ranalletta M. Partial epiphyseal preservation and intercalary allograft reconstruction in high-grade metaphyseal osteosarcoma of the knee. J Bone Joint Surg Am. 2004;86(12):2686-93.

12. Sakayama K, Kidani T, Fujibuchi T, Kamogawa J, Yamamoto H, Shibata T. Reconstruction surgery for patients with musculoskeletal tumor, using a pasteurized autogenous bone graft. Int J Clin Oncol. 2004;9(3):167-73.

13. Chang DW, Weber KL. Use of a vascularized fibula bone flap and intercalary allograft for diaphyseal reconstruction after resection of primary extremity bone sarcomas. Plast Reconstr Surg. 2005;116(7):1918-25.

14. Chen TH, Chen WM, Huang CK. Reconstruction after intercalary resection of malignant bone tumours: comparison between segmental allograft and extracorporeally-irradiated autograft. J Bone Joint Surg Br. 2005;87(5):704-9.

15. Deijkers RL, Bloem RM, Kroon HM, Van Lent JB, Brand R, Taminiau AH. Epidiaphyseal versus other intercalary allografts for tumors of the lower limb. Clin Orthop Relat Res. 2005;439:151-60.

16. Pollock R, Stalley P, Lee K, Pennington D. Free vascularized fibula grafts in limb-salvage surgery. J Reconstr Microsurg. 2005;21(2):79-84.

17. Moran SL, Shin AY, Bishop AT. The use of massive bone allograft with intramedullary free fibular flap for limb salvage in a pediatric and adolescent population. Plast Reconstr Surg. 2006;118(2):413-9.

18. Muramatsu K, Ihara K, Doi K, Shigetomi M, Hashimoto T, Taguchi T. Reconstruction of massive femur defect with free vascularized fibula graft following tumor resection. Anticancer Res. 2006;26(5b):3679-83.

19. Capanna R, Campanacci DA, Belot N, et al. A new reconstructive technique for intercalary defects of long bones: the association of massive allograft with vascularized fibular autograft. Long-term results and comparison with alternative techniques. Orthop Clin North Am. 2007;38(1):51-60, vi.

20. Jeon D-G, Kim MS, Cho WH, Song WS, Lee S-Y. Pasteurized autograft for intercalary reconstruction: an alternative to allograft. Clinical orthopaedics and related research. 2007;456:203-10.

21. Krieg AH, Davidson AW, Stalley PD. Intercalary femoral reconstruction with extracorporeal irradiated autogenous bone graft in limb-salvage surgery. J Bone Joint Surg Br. 2007;89(3):366-71.

22. Sugiura H, Takahashi M, Nakanishi K, Nishida Y, Kamei Y. Pasteurized intercalary autogenous bone graft combined with vascularized fibula. Clinical orthopaedics and related research. 2007;456:196-202.

23. Muscolo DL, Ayerza MA, Aponte-Tinao L, Farfalli G. Allograft reconstruction after sarcoma resection in children younger than 10 years old. Clinical orthopaedics and related research. 2008;466(8):1856-62.

24. Abed YY, Beltrami G, Campanacci DA, Innocenti M, Scoccianti G, Capanna R. Biological reconstruction after resection of bone tumours around the knee: long-term follow-up. J Bone Joint Surg Br. 2009;91(10):1366-72.

25. Innocenti M, Abed YY, Beltrami G, Delcroix L, Manfrini M, Capanna R. Biological reconstruction after resection of bone tumors of the proximal tibia using allograft shell and intramedullary free vascularized fibular graft: long-term results. Microsurgery. 2009;29(5):361-72.

26. Wang Z, Guo Z, Li X, et al. Limb salvage surgery for malignant bone tumors of the extremities in children and adolescents. Chinese-German Journal of Clinical Oncology. 2009;8(11):631-7.

27. Zimel MN, Cizik AM, Rapp TB, Weisstein JS, Conrad EU, 3rd. Megaprosthesis versus Condyle-sparing intercalary allograft: distal femoral sarcoma. Clinical orthopaedics and related research. 2009;467(11):2813-24.

28. Hariri A, Mascard E, Atlan F, et al. Free vascularised fibular graft for reconstruction of defects of the lower limb after resection of tumour. J Bone Joint Surg Br. 2010;92(11):1574-9.

29. Jager T, Journeau P, Dautel G, Barbary S, Haumont T, Lascombes P. Is combining massive bone allograft with free vascularized fibular flap the children's reconstruction answer to lower limb defects following bone tumour resection? Orthop Traumatol Surg Res. 2010;96(4):340-7.

30. Li J, Wang Z, Guo Z, Chen G-J, Fu J, Pei G-X. The use of allograft shell with intramedullary vascularized fibula graft for intercalary reconstruction after diaphyseal resection for lower extremity bony malignancy. J Surg Oncol. 2010;102(5):368-74.

31. Yang YF, Zhang GM, Xu ZH, Wang JW. Homeochronous usage of structural bone allografts with vascularized fibular autografts for biological repair of massive bone defects in the lower extremities after bone tumor excision. J Reconstr Microsurg. 2010;26(2):109-15.

32. Brunet O, Anract P, Bouabid S, et al. Intercalary defects reconstruction of the femur and tibia after primary malignant bone tumour resection. A series of 13 cases. Orthopaedics & traumatology, surgery & research : OTSR. 2011;97(5):512-9.

33. Li J, Wang Z, Guo Z, Chen G-j, Li S-w, Pei G-X. The use of massive allograft with intramedullary fibular graft for intercalary reconstruction after resection of tibial malignancy. J Reconstr Microsurg. 2011;27(1):37-46.

34. Puri A, Gulia A. Paediatric diaphyseal malignant tumors: options for reconstruction after intercalary resection. J Pediatr Orthop B. 2011;20(5):309-17.

35. Aponte-Tinao L, Farfalli GL, Ritacco LE, Ayerza MA, Muscolo DL. Intercalary femur allografts are an acceptable alternative after tumor resection. Clin Orthop Relat Res. 2012;470(3):728-34.

36. Farfalli GL, Aponte-Tinao L, Lopez-Millán L, Ayerza MA, Muscolo DL. Clinical and functional outcomes of tibial intercalary allografts after tumor resection. Orthopedics. 2012;35(3):e391-e6.

37. Frisoni T, Cevolani L, Giorgini A, Dozza B, Donati DM. Factors affecting outcome of massive intercalary bone allografts in the treatment of tumours of the femur. J Bone Joint Surg Br. 2012;94(6):836-41.

38. Mottard S, Grimer RJ, Abudu A, et al. Biological reconstruction after excision, irradiation and reimplantation of diaphyseal tibial tumours using an ipsilateral vascularised fibular graft. J Bone Joint Surg Br. 2012;94(9):1282-7.

39. Sugiura H, Nishida Y, Nakashima H, Yamada Y, Tsukushi S, Yamada K. Evaluation of long-term outcomes of pasteurized autografts in limb salvage surgeries for bone and soft tissue sarcomas. Archives of orthopaedic and trauma surgery. 2012;132(12):1685-95.

40. Tanaka K, Maehara H, Kanaya F. Vascularized fibular graft for bone defects after wide resection of musculoskeletal tumors. Journal of orthopaedic science : official journal of the Japanese Orthopaedic Association. 2012;17(2):156-62.

41. Nakamura T, Abudu A, Grimer RJ, Carter SR, Jeys L, Tillman RM. The clinical outcomes of extracorporeal irradiated and re-implanted cemented autologous bone graft of femoral diaphysis after tumour resection. International orthopaedics. 2013;37(4):647-51.

42. Niethard M, Tiedke C, Andreou D, et al. Bilateral fibular graft: biological reconstruction after resection of primary malignant bone tumors of the lower limb. Sarcoma. 2013;2013:205832.

43. Rabitsch K, Maurer-Ertl W, Pirker-Frühauf U, Wibmer C, Leithner A. Intercalary reconstructions with vascularised fibula and allograft after tumour resection in the lower limb. Sarcoma. 2013;2013:160295.

44. Umer M, Umer HM, Qadir I, et al. Autoclaved tumor bone for skeletal reconstruction in paediatric patients: A low cost alternative in developing countries. BioMed Research International. 2013;2013.

45. Bus MPA, Dijkstra PDS, van de Sande MAJ, et al. Intercalary allograft reconstructions following resection of primary bone tumors: a nationwide multicenter study. J Bone Joint Surg Am. 2014;96(4):e26-e.

46. Han I, Kim JH, Cho HS, Kim HS. Low-heat treated autograft versus allograft for intercalary reconstruction of malignant bone tumors. J Surg Oncol. 2014;110(7):823-7.

47. Igarashi K, Yamamoto N, Shirai T, et al. The long-term outcome following the use of frozen autograft treated with liquid nitrogen in the management of bone and soft-tissue sarcomas. The bone & joint journal. 2014;96-B(4):555-61.

48. Shin SH, Lee K-H, Jang SP, Mun G-H, Seo SW. Massive intercalary reconstruction of lower limb after wide excision of malignant tumors: an alternative to amputation or rotationplasty. J Reconstr Microsurg. 2014;30(4):255-62.

49. Aponte-Tinao L, Ayerza MA, Muscolo DL, Farfalli GL. Survival, recurrence, and function after epiphyseal preservation and allograft reconstruction in osteosarcoma of the knee. Clin Orthop Relat Res. 2015;473(5):1789-96.

50. Erol B, Basci O, Topkar MO, Caypinar B, Basar H, Tetik C. Mid-term radiological and functional results of biological reconstructions of extremity-located bone sarcomas in children and young adults. J Pediatr Orthop B. 2015;24(5):469-78.

51. Ogura K, Miyamoto S, Sakuraba M, Fujiwara T, Chuman H, Kawai A. Intercalary reconstruction after wide resection of malignant bone tumors of the lower extremity using a composite graft with a devitalized autograft and a vascularized fibula. Sarcoma. 2015;2015:861575-.

52. Qu H, Guo W, Yang R, et al. Reconstruction of segmental bone defect of long bones after tumor resection by devitalized tumor-bearing bone. World J Surg Oncol. 2015;13:282-.

53. Weichman KE, Dec W, Morris CD, Mehrara BJ, Disa JJ. Lower Extremity Osseous Oncologic Reconstruction with Composite Microsurgical Free Fibula Inside Massive Bony Allograft. Plast Reconstr Surg. 2015;136(2):396-403.

54. Houdek MT, Wagner ER, Stans AA, et al. What Is the Outcome of Allograft and Intramedullary Free Fibula (Capanna Technique) in Pediatric and Adolescent Patients With Bone Tumors? Clin Orthop Relat Res. 2016;474(3):660-8.

55. Li J, Zhang F, Yang M, et al. Extracorporeally frozen tumour-bearing bone combined with free vascularised fibula for the intercalary reconstruction of femoral defect after resection of bony sarcoma. J Plast Reconstr Aesthet Surg. 2016;69(6):856-63.

56. Emori M, Kaya M, Irifune H, et al. Vascularised fibular grafts for reconstruction of extremity bone defects after resection of bone and soft-tissue tumours. Bone and Joint Journal. 2017;99B(9):1237-43.

57. Gupta S, Kafchinski LA, Gundle KR, et al. Intercalary allograft augmented with intramedullary cement and plate fixation is a reliable solution after resection of a diaphyseal tumour. The bone & joint journal. 2017;99-B(7):973-8.

58. Manfrini M, Bindiganavile S, Say F, et al. Is There Benefit to Free Over Pedicled Vascularized Grafts in Augmenting Tibial Intercalary Allograft Constructs? Clinical orthopaedics and related research. 2017;475(5):1322-37.

59. Zekry KM, Yamamoto N, Hayashi K, et al. Intercalary frozen autograft for reconstruction of malignant bone and soft tissue tumours. Int Orthop. 2017;41(7):1481-7.

60. Aponte-Tinao LA, Albergo JI, Ayerza MA, Muscolo DL, Ing FM, Farfalli GL. What Are the Complications of Allograft Reconstructions for Sarcoma Resection in Children Younger Than 10 Years at Long-term Followup? Clin Orthop Relat Res. 2018;476(3):548-55.

61. Campanacci DA, Totti F, Puccini S, et al. Intercalary reconstruction of femur after tumour resection: is a vascularized fibular autograft plus allograft a long-lasting solution? The bone & joint journal. 2018;100-B(3):378-86.

62. Houdek MT, Rose PS, Milbrandt TA, Stans AA, Moran SL, Sim FH. Comparison of Pediatric Intercalary Allograft Reconstructions with and without a Free Vascularized Fibula. Plast Reconstr Surg. 2018;142(4):1065-71.

63. Ikuta K, Nishida Y, Sugiura H, et al. Predictors of complications in heat-treated autograft reconstruction after intercalary resection for malignant musculoskeletal tumors of the extremity. J Surg Oncol. 2018;117(7):1469-78.

64. Lee SY, Jeon D-G, Cho WH, Song WS, Kim BS. Are Pasteurized Autografts Durable for Reconstructions After Bone Tumor Resections? Clinical orthopaedics and related research. 2018;476(9):1728-37.

65. Puri A, Byregowda S, Gulia A, Patil V, Crasto S, Laskar S. Reconstructing diaphyseal tumors using radiated (50 Gy) autogenous tumor bone graft. J Surg Oncol. 2018;118(1):138-43.

66. Ghoneimy AME, Sherbiny ME, Kamal N. Use of Vascularized Fibular Free Flap in the Reconstruction of the Femur in Pediatric and Adolescent Bone Sarcomas: Complications and Functional Outcome. J Reconstr Microsurg. 2019;35(2):156-62.

67. Krieg AH, Lenze U, Schultze L, Gross MW, Haug M. Extracorporeal Irradiation and Reimplantation of Tumor-bearing Bone Segments Following Diaphyseal Sarcoma Resection at the Tibia. Anticancer Res. 2019;39(4):2015-23.

68. Li J, Chen G, Lu Y, Zhu H, Ji C, Wang Z. Factors Influencing Osseous Union Following Surgical Treatment of Bone Tumors with Use of the Capanna Technique. J Bone Joint Surg Am. 2019:10.2106/JBJS.19.00380.

69. Liu T, Ling L, Zhang Q, Liu Y, Guo X. Evaluation of the Efficacy of Pasteurized Autograft and Intramedullary Vascularized Fibular Transfer for Osteosarcoma of the Femoral Diaphysis. Orthop Surg. 2019;11(5):826-34.

70. Oike N, Kawashima H, Ogose A, et al. Long-term outcomes of an extracorporeal irradiated autograft for limb salvage operations in musculoskeletal tumours: over ten years’ observation. The bone & joint journal. 2019;101-B(9):1151-9.

71. Ruiz-Moya A, Lagares-Borrego A, Sicilia-Castro D, et al. Pediatric extremity bone sarcoma reconstruction with the vascularized fibula flap: Observational study assessing long-term functional outcomes, complications, and survival. J Plast Reconstr Aesthet Surg. 2019;72(12):1887-99.

72. Salunke AA, Shah J, Chauhan TS, et al. Reconstruction with biological methods following intercalary excision of femoral diaphyseal tumors. J Orthop Surg (Hong Kong). 2019;27(1):2309499018822242-.

73. Shemesh SS, Pretell-Mazzini J, Quartin PAJ, Rutenberg TF, Conway SA. Surgical treatment of low-grade chondrosarcoma involving the appendicular skeleton: long-term functional and oncological outcomes. Archives of orthopaedic and trauma surgery. 2019;139(12):1659-66.

74. Albergo JI, Gaston LC, Farfalli GL, et al. Failure rates and functional results for intercalary femur reconstructions after tumour resection. Musculoskelet Surg. 2020;104(1):59-65.

75. Mihara A, Muramatsu K, Hashimoto T, Iwanaga R, Ihara K, Sakai T. Combination of Extracorporeally-irradiated Autograft and Vascularized Bone Graft for Reconstruction of Malignant Musculoskeletal Tumor. Anticancer Res. 2020;40(3):1637-43.

76. Sanders PTJ, Spierings JF, Albergo JI, et al. Long-Term Clinical Outcomes of Intercalary Allograft Reconstruction for Lower-Extremity Bone Tumors. J Bone Joint Surg Am. 2020.
